# Epigenetics and its role in effecting agronomical traits

**DOI:** 10.3389/fpls.2022.925688

**Published:** 2022-08-15

**Authors:** Chainika Gupta, Romesh K. Salgotra

**Affiliations:** School for Biotechnology, Sher-e-Kashmir University of Agricultural Sciences & Technology of Jammu, Jammu, Jammu and Kashmir, India

**Keywords:** epigenetic, epialleles, agronomic traits, plant species, improvement

## Abstract

Climate-resilient crops with improved adaptation to the changing climate are urgently needed to feed the growing population. Hence, developing high-yielding crop varieties with better agronomic traits is one of the most critical issues in agricultural research. These are vital to enhancing yield as well as resistance to harsh conditions, both of which help farmers over time. The majority of agronomic traits are quantitative and are subject to intricate genetic control, thereby obstructing crop improvement. Plant epibreeding is the utilisation of epigenetic variation for crop development, and has a wide range of applications in the field of crop improvement. Epigenetics refers to changes in gene expression that are heritable and induced by methylation of DNA, post-translational modifications of histones or RNA interference rather than an alteration in the underlying sequence of DNA. The epigenetic modifications influence gene expression by changing the state of chromatin, which underpins plant growth and dictates phenotypic responsiveness for extrinsic and intrinsic inputs. Epigenetic modifications, in addition to DNA sequence variation, improve breeding by giving useful markers. Also, it takes epigenome diversity into account to predict plant performance and increase crop production. In this review, emphasis has been given for summarising the role of epigenetic changes in epibreeding for crop improvement.

## Introduction

The increase in the world’s population at an alarming rate as well as the problems posed by global climate change on crop production, urges us to improve agricultural yield and quality in a sustainable manner (Takeda and Matsuoka, 2008; Fedoroff, 2010). This goal can be achieved by genetic crop improvement, which integrates various fields like plant physiology, genetics and biotechnology. The incorporation of genetic material in to a crop of interest is one of its most important prerequisites (Lombardo et al., 2016). Besides genetic variation, a better knowledge of the influence of epigenetic changes on plant phenotype has allowed the crop development process to move even faster (Merce et al., 2020). The word epigenetics was given by Waddington, combining the phrases “epigenesis” and “genetics.” He defined epigenetics as “the study of the interaction between a gene and its product, that produces the phenotype” (Waddington, 1942). Plant developmental processes and phenotypic plasticity, particularly adaptive responses to environmental challenges, are influenced by epigenetic information. This has prompted scientists to reconsider the relationship between genotypes and phenotypes (Pecinka and Mittelsten Scheid, 2012; Springer, 2013; Zhang Y. Y. et al., 2013; Baulcombe and Dean, 2014; Pikaard and Mittelsten Scheid, 2014; Meyer, 2015; Rey et al., 2016). Epigenetic variations have been assessed and quantified with their effect on not only plant developmental processes and plant responses to environmental constraints (Baulcombe and Dean, 2014; Cortijo et al., 2014; Meyer, 2015; Takatsuka and Umeda, 2015) but also important agronomical traits like respiration, energy-use efficiency, yield components and seed quality (Hauben et al., 2009; Shi et al., 2009; Long et al., 2011; Chen and Zhou, 2013). At the molecular level, a gene’s transcription is influenced by its DNA sequence as well as how genes are organised within the chromosome’s intricate architecture. The DNA of eukaryotes is extremely condensed and closely connected with proteins called histones and this combination is referred to as chromatin. To start transcription at a given gene, the chromatin at that location must be open for binding of transcription factors (TF) as well as RNA polymerase. Hence, whether a gene is “on” or “off” is determined by the chromatin state at a specific gene. The accessibility of chromatin to the transcriptional machinery is influenced by a variety of mechanisms like methylation of DNA, posttranslational modifications of histone, chromatin remodelling as well as non-coding RNAs. The chromatin state and expression pattern of genes that have been created can be conserved through numerous generations, thus called epigenetic.

**FIGURE 1:**
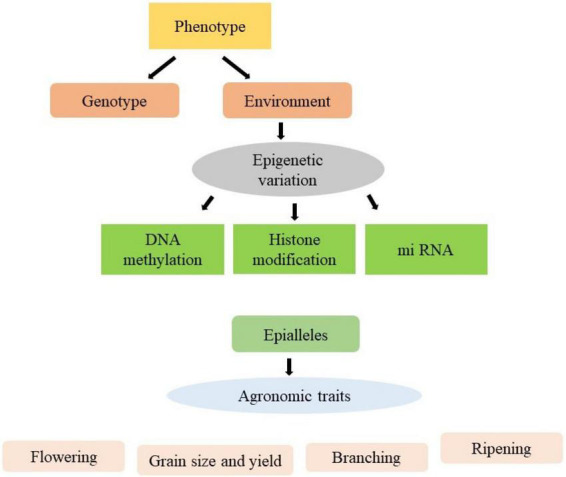
Use of epialleles for improvement of various agronomical traits.

The environmental changes may result in heritable epigenetic changes which are associated with changes in gene expression and variation in phenotype (Kakoulidou et al., 2021). These phenotypic variations are contributing to epibreeding for the improvement of various crop production traits (Figure 1). Although the epigenetic mechanism are well established in model plants, the emphasis has been given to the improvement of phenotypic traits of crops. The epigenetic modifications may be used as markers to allow the use of epigenome diversity in crop improvement programmes. No doubt various difficulties are arising in transferring the epigenetic system from model plants to crops, however numerous high-throughput technologies have been evolved to solve these problems. The epigenetic modifications influence gene expression by changing the state of chromatin, which underpins plant growth and dictates phenotypic responsiveness with respect to extrinsic and intrinsic inputs. Epigenetic modifications, in addition to DNA sequence variation, improve breeding by giving useful markers. Also, it takes epigenome diversity into account to predict plant performance and increase crop production. Plant epibreeding or the utilisation of epigenetic variation for crop development, has a wide range of applications in the field of crop improvement. Epigenetics is the study of changes in gene expression that are heritable and caused by methylation of DNA, post-translational modifications of histones or RNA interference rather than an alteration in the underlying sequence of DNA (Kapazoglou et al., 2018). Epigenetic variants of agronomic importance have been identified in rice (dwarf phenotype), apple (anthocyanin production), oilseed rape (decreased oil content), pigeon pea (high heterosis), pineapple (increased somatic embryogenesis), soybean (enhanced yield and stability), melon (sex determination), and tomato (fruit ripening). The main challenge faced by agriculture in the present century is to boost agricultural productivity. Epigenetics provides essential biological information that could be directly used to enhance crop tolerance and adaptability (Kakoulidou et al., 2021). Keeping in perspective the importance of epigenetic changes in improving the crop species, the present review summarises the cutting-edge knowledge comprehensively on the epigenetic molecular aspects and their utilisation in crop breeding. Moreover, it also presents acumens gleaned on the improvement of agronomic traits of important crops.

## Molecular epigenetic mechanisms

### Methylation of DNA

DNA methylation is a heritable process where a methyl group (CH3) is added to 5th carbon of a cytosine base. It governs a variety of functions like an expression of a gene, genomic stability, gene imprinting, inactivation of transposable elements and its disruption can result in developmental abnormalities such as failure in tomato fruit ripening (Baulcombe and Dean, 2014; Iwasaki and Paszkowski, 2014; Lang et al., 2017; Zhang H. et al., 2018; Gallego-Bartolome, 2020). It is passed on in a mendelian fashion (Niederhuth and Schmitz, 2014). However, in paramutation, methylation variations can generate phenotypic diversity because of spontaneous methylation alterations that result in non-mendelian inheritance (Weigel and Colot, 2012). Novel epialleles may result from variations in DNA methylation patterns, which may aid plant improvement and adaptation. Heavy methylation of transposable elements and other repetitive DNA sequences in heterochromatin has been observed in *Arabidopsis thaliana* during a genome-wide DNA methylation study (Zhang et al., 2006; Henderson and Jacobsen, 2007). Euchromatic chromosomal arms include interspersed transposon-associated DNA methylation (Zhang et al., 2006). Symmetric and asymmetric methylation of cytosine bases also occurs (Henderson and Jacobsen, 2007). The former denotes the presence of cytosine that is methylated in double stranded DNA, whereas the latter denotes the presence of cytosine that is methylated in single-stranded DNA only (Mbichi et al., 2020).

The methylation pattern is usually CG (symmetric), CHG (symmetric) or CHH (H = a nucleotide other than G, asymmetric). It is particularly abundant in heterochromatic transposable elements (TEs) and repetitions. When DNA methylation occurs in gene regulatory areas, it can cause Transcriptional Gene Silencing (TGS). It has been discovered that the DNA methyl-readers SU(VAR)3-9 homologs SUVH1 and SUVH3 have a role in enhancing gene expression in some cases (Harris et al., 2018; Xiao et al., 2019).

DNA methylation is further categorised into

1. *De novo* methylation
2. maintenance methylation

*De novo* methylation occurs when previously unmethylated cytosine residues are methylated, resulting in the formation of novel methylation patterns. On the other hand, in maintenance methylation, previous existing methylation patterns are retained after DNA replication (Chen and Li, 2004). The enzymes methyltransferases and demethylases control DNA methylation. The methylation of CG and CHG is carried out by METHYLTRANSFERASE 1 (MET1) and CHROMOMETHYLASE 3 (CMT3), respectively (Zhang H. et al., 2018). Two pathways create *De novo* CHH methylation. The first one involves RNA-dependent DNA methylation. Here, the small interfering RNAs (siRNAs) are targeted to homologous genomic loci by members of the ARGONAUTE (AGO) family and then methylated by DOMAINS REARRANGED METHYLTRANSFERASE2 (DRM2). The second one involves histone H1-rich chromatic areas, where CHROMOMETHYLASE 2 (CMT2) interacts with DECREASE IN DNA METHYLATION1 (DDM1) (Zemach et al., 2013). The methylation in DNA is eliminated by a process involving active or passive demethylation. Active demethylation is mediated by DNA glycosylases like Arabidopsis DEMETER (DME) and REPRESSOR OF SILENCING 1 (ROS1), which play a significant role in the gene expression control. Passive demethylation mainly occurs during DNA replication (Gehring et al., 2009; Zhu, 2009; Tang et al., 2016).

The accessibility of genomic areas to regulatory proteins/protein complexes is influenced by cytosine methylation, which alters chromatin structure and the rate of gene transcription. When found in the promoter and enhancer regions, it has been linked to gene repression (Charlet et al., 2016). However, in the case of gene body methylation (gbM), 5-mC in the transcribed region has the potential to supress or increase transcription (Buck-Koehntop and Defossez, 2013; Spruijt and Vermeulen, 2014). Due to its heredity and potential to influence plant phenotypes, methylation of DNA contributes to crop productivity. The traits of agronomic importance including time of flowering, dormancy in seed, yield, etc., are influenced by the methylation of DNA (Liu and Wendel, 2003; Zhang Y. Y. et al., 2013; Song et al., 2017).

### Mechanism of DNA methylation

The DNA methylation in plants takes place through the canonical RNA-directed DNA methylation (RdDM) pathway as well as non-canonical RdDM pathways. The RNA polymerase IV (Pol IV) synthesises single-stranded RNAs (ssRNAs), which get transformed into double-stranded RNAs (dsRNAs) with the help of RNA-DEPENDENT RNA POLYMERASE 2 (RDR2), in the canonical RdDM pathway. DICER-LIKE 3 (DCL3) cuts these dsRNAs into small interfering RNAs (siRNAs) of 24-nucleotides in length, which get integrated into ARGONAUTE 4 (AGO4) and ARGONAUTE 6 (AGO6) (Zhang H. et al., 2018). The recruitment of Pol IV to chromatin requires chromatin remodelers like SAWADEE HOMEODOMAIN HOMOLOG 1 (SHH1) and the CLASSY family (Zhang H. et al., 2018; Zhou et al., 2018).

Small RNAs (sRNAs) (from viruses and Pol II transcripts) guide RdDM in non-canonical RdDM pathways (Cuerda-Gil and Slotkin, 2016). The dsRNAs are sliced into 21–24 nucleotide sRNAs by different DCL proteins followed by their incorporation into AGO proteins. The complex binds to complementary RNA and can cleave it or repress translation leading to post-transcriptional gene silencing (PTGS) (Martinez de Alba et al., 2013). When sRNAs are incorporated into AGO4/AGO6, they can cause complementary DNA sequences to be methylated by Pol V and DRM2, potentially leading to the silencing of genes (Cuerda-Gil and Slotkin, 2016).

### MicroRNAs

MicroRNAs (miRNAs) are short non-coding RNA encoded by miRNA genes. They are of 20–24 nucleotides long (Jones-Rhoades et al., 2006). They can influence complex biological processes in plants by regulating gene expression (Chen, 2009; Voinnet, 2009; Pantaleo et al., 2010; Sun, 2012; Djami-Tchatchou and Dubery, 2015). They bind to messenger RNAs with partially complementary sequences (mRNAs). miRNA genes are found in intergenic regions as well as within introns, while some of them are found in clusters throughout the genome and transcribed as long polycistronic RNAs (Djami-Tchatchou et al., 2017).

miRNA biogenesis takes place in the nucleus (Bartel, 2004; Jones-Rhoades et al., 2006). RNA polymerase II is commonly used to transcribe miRNA genes, leading to the formation of primary miRNA (pri-miRNA) (Xie et al., 2005; Pantaleo et al., 2010). It is converted into precursor miRNA (pre-miRNA) with a self-complementary stem-loop on which the Dicer-like 1 (DCL1) enzyme acts. It dices the pre-miRNA in the presence of HYPONASTIC LEAVES 1 (HYL1) and Serrate (SE) and miRNA: miRNA duplex is formed (Tang and Chu, 2017). HUA ENHANCER 1 (HEN1) methylates the duplex which is transported to the cytoplasm by EXPORTIN5, HASTY (Park et al., 2005; Pelaez et al., 2012). The duplexes are then placed in the RNA-induced silencing complex (RISC), which contains the ARGONAUTE (AGO) proteins. After RISC loading, AGO1 unwinds miRNA: miRNA duplexes with one strand sent to the exosome for degradation and the other strand is inserted into the RISC complex (Sun, 2012). The mature miRNA directs RISC to target complementary mRNAs leading to their cleavage or translational repression (Sun, 2012).

Most miRNAs work as negative regulators of their target transcripts. They use considerable sequence complementarity to target coding sequences (ORFs). Translational repression occurs in targets with low sequence complementarity. It has been reported that pairing of bases at the initial 2–13 nucleotides of miRNA towards the 5′ end is important because many targets suffer AGO-mediated cleavage at positions 9–11 (Axtell, 2013). Mismatches around the 3′ end of miRNAs are thought to be less “destructive” than mismatches near the 5′ end or in the core regions (Liu N. et al., 2014). Although perfect complementarity between miRNA targets is uncommon, mismatches have been observed frequently at either the extreme 5′ end of miRNAs or toward 3′. In some cases, the interaction of miRNA with a target is complementary at two ends of the region, with a bulge or mismatch in the center area (Pandey et al., 2019). The targets of miRNA are transcription factors (TFs) which are the important regulators of plant development. These include

(i) SQUAMOSA-promoter binding protein-like (SPL) TFs: It plays a role in phase transition or vegetative and reproductive development and is negatively regulated by miR156, miR157, or miR529 (Chuck et al., 2010; Miura et al., 2010; Bhogale et al., 2014; Ferreira e Silva et al., 2014; Chen et al., 2015; Wang et al., 2015).
(ii) APETALA2 (AP2) family of TFs: They are inhibited by miR172 and impact phase transition, organ development and sex determination (Martin et al., 2009a; Houston et al., 2013; Lee et al., 2014; Wang et al., 2015).
(iii) TEOSINTE BRANCHED/CYCLOIDEA/PCF (TCP) TFs: These are regulated by miR319, which impacts tolerance to cold, defence, and morphogenesis in leaves (Ori et al., 2007; Yang et al., 2013; Zhao et al., 2015; Zhang C. et al., 2016).
(iv) MYB TFs: miR159 antagonised these TFs and plays a role in the development of flowers and response to heat or acts along with miR828 in controlling the modulation of fibre growth (Tsuji et al., 2006; Wang et al., 2012; Guan et al., 2014).
(v) NAC TFs: These are expressed by genes targeted by miR164 and are involved in the growth of lateral roots, tolerance to drought and immunological response (Li et al., 2012; Fang et al., 2014; Feng et al., 2014).
(vi) Growth-regulating factors (GRFs): These are controlled by miR396 that regulates the size of grain and yield, development of inflorescence and tolerance to salt–alkali stress (Hewezi et al., 2012; Liu N. et al., 2014; Che et al., 2015; Duan et al., 2015; Gao et al., 2015; Hu et al., 2015).
(vii) Auxin response factors (ARFs): These are responsible for response to auxin, defence responses as well as crucial in developmental stages and are targeted by miR160 or miR167 (Li et al., 2014; Liu N. et al., 2014; Nizampatnam et al., 2015; Wang et al., 2015; Damodharan et al., 2016; Huang et al., 2016).
(viii) DNA-binding with one finger (Dof) TF RDD1, SCARECROW-like (SCL) TF HvSCL, zinc finger domain-containing TF GhCHR, Timing of CAB Expression 1 TF TaTOC1 and the HD-ZIPIII family member ROLLED LEAF1 (RLD1): miR166, miR171, miRNVL5, miR408, and miR166 are all negative regulators of these genes. These are involved in the uptake of ions, determination of floral meristem, salt stress response, and flowering time (Juarez et al., 2004; Curaba et al., 2013; Gao et al., 2016; Iwamoto and Tagiri, 2016; Zhao et al., 2016).

### Histone

Genomic DNA in eukaryotes is firmly condensed with proteins to form chromatin, which impacts the accessibility of transcription factors and cofactors for modulating gene expression. The most fundamental unit of chromatin is the nucleosome, which consists of DNA wind around a histone octamer (two copies of four histone proteins viz H2A, H2B, H3, and H4) (Luger et al., 1997; Liu et al., 2016). Histone H1 maintains this structure by binding to the nucleosome along with sections including linker DNA. The availability of chromatin to the transcriptional machinery is influenced by DNA methylation, posttranslational histone modifications, histone variant exchange, chromatin remodelling, and the inclusion of non-coding RNAs. Histone proteins undergo post translational modifications (PTMs). Most histone modification sites are found in histone N-terminal tails and include acetylation, methylation, phosphorylation, ubiquitination, and sumoylation (Millar and Grunstein, 2006; Lauria and Rossi, 2011; Yuan et al., 2013; Liu X. et al., 2014). Due to the presence of high lysine and arginine content in histones, their amino (N-terminal) tails are extremely basic. These tails protrude from the nucleosome core, making protein–protein and protein–DNA interactions possible. Reversible covalent changes occur at these histone tails. These changes act as “histone code,” indicating how chromatin functions and transcriptional activities are carried out (Jenuwein and Allis, 2001). These alterations can influence chromatin shape and transcription of genes by changing the interface linking histones and DNA as well as modifying the attachment of regulatory proteins to DNA (Pikaard and Mittelsten Scheid, 2014; Allis and Jenuwein, 2016; Chang et al., 2019). The activation or repression of transcription is linked to histone modifications.

#### Histone methylation

It is required for biological processes like transcriptional control and the formation of heterochromatin. Histone methylation leads to retention of the charge on amino acids and does not affect electrostatic properties of histones (Ueda and Seki, 2020). It occurs mainly at the lysine and arginine residues of H3 and H4 histones resulting in alteration in gene expression that has both activating and repressive effects (Swygert and Peterson, 2014). The lysine residues can be monomethylated, dimethylated or trimethylated (Cloos et al., 2008). Different methylation has effects on gene expression in different ways (Liu et al., 2010; Zhao et al., 2019). In the case of *A*. *thaliana*, trimethylation of Lys 27 (H3K27me3) suppresses gene expression while trimethylation of Lys 4 (H3K4me3) increases gene transcription (Berr et al., 2011; Zheng and Chen, 2011). The enzymes that carry out addition, removal or reading out of methylation marks are known as writers, erasers and readers. Writers are the enzymes that add posttranslational modifications to a protein. Erasers are the enzymes that remove a protein’s posttranslational modification. Readers are the proteins that recognise and bind to a post-translationally modified substrate (Xu et al., 2017). The enzymes which carry out the transfer of methyl groups to histones are histone methyltransferases (HMTs). They comprise of histone lysine methyltransferases (HKMTs) and protein arginine methyltransferases (PRMTs). Histone methyltransferases contain the Set (Suppressor of Variegation 3–9, Enhancer of zeste, and Trithorax) Domain, belong to the gene family SDG, and add lysine methylation to histones. In Arabidopsis, 37 SDG genes have been discovered (Thorstensen et al., 2011). The action of many HKMTs bring about lysine methylation, primarily at K4, K9, K27, and K36 of H3 along with K 20 of H4. This changes their interaction with reading proteins, causing structural changes in chromatin that result in transcription activation or repression (Teperino et al., 2010). The methylation in H3K4 and H3K36 can lead to transcriptional activation, whereas H3K9 and H3K27 methylation can lead to transcriptional repression (Ueda and Seki, 2020). Two types of histone demethylases catalyse the removal of histone lysine methylation: Jumonji C (JmjC) domain-containing proteins and Lysine-Specific Demethylase (LSD)-like proteins. Jumonji-C (JmjC) domain–containing proteins remove methyl groups from dimethylated and trimethylated lysines preferentially while Lysine-specific demethylases (LSDs) demethylate monomethylated and dimethylated lysines (Shi et al., 2004; Tsukada et al., 2006). H3 and H4 arginine [R] residues can also be methylated. Arginine methylation can be symmetric or asymmetric and it has been observed in R17 (of H3) and R3 (of H4) (Ueda and Seki, 2020). Methylation of arginine could give an opposing outcome depending on whether it is symmetric or asymmetric. For example, H4R3me2a (asymmetric) is an activation mark while H4R3me2s (symmetric) is a repression mark.

#### Histone acetylation and deacetylation

Acetylation on histones is a flexible and dynamic process. It is correlated with transcription activity (Allfrey et al., 1964). The amino terminal tails of histones contain lysine and arginine (Luger and Richmond, 1998). The transfer of an acetyl group to the ε-amine group of the N-terminal lysine residue of histone protein is known as histone acetylation (Pandey et al., 2002; Kouzarides, 2007). It neutralises the positive charge of the histone tails and enhances hydrophobicity (Allis and Jenuwein, 2016; Onufriev and Schiessel, 2019). As a result, the affinity of histone proteins for negatively charged DNA decreases and the chromatin state switches from a closed to an open state (Clayton et al., 2006; Li S. et al., 2019). This leads to the initiation of transcription by attachment of RNA polymerase along with transcription factors to the promoter region of the gene. It has been discovered that an increase in histone acetylation near the transcription start site is positively linked with gene expression (Srivastava et al., 2014; Wang et al., 2017; Kim et al., 2020).

Histone acetylation is mediated by histone acetyltransferases (HATs) and deacetylation by histone deacetylases (HDACs) (Pandey et al., 2002; Kumar et al., 2021). HATs activate genes by adding acetyl groups to lysine residues in histone N-terminal tails as well as globular domains (Bjerling et al., 2002; Pandey et al., 2002; Iwasaki et al., 2011; Pradeepa et al., 2016). HDACs, on the other hand, remove these acetyl groups from histones leading to an increase in interaction between DNA and histones. These counteract the effects of HATs resulting in gene repression (Gallinari et al., 2007; Gu et al., 2017; Yang et al., 2020). HATs and HDACs mainly target lysine residues in histones, such as H3K9, H3K14, H3K36, H4K5, H4K8, H4K12, and H4K16 (Bjerling et al., 2002).

Based on their subcellular distribution, HATs are categorised as: Type A HAT and Type B HAT. The type A HATs are engaged in the acetylation of nuclear histone and consequently regulate chromatin assembly and transcription of genes (Carrozza et al., 2003). It includes GCN5-related acetyltransferases (GNATs), MYST (for MOZ, Ybf2/Sas3, Sas2, and Tip60)-related HATs, p300/CBP HATs and the TFIID subunit TAF250 (Sterner and Berger, 2000; Pandey et al., 2002; Carrozza et al., 2003; Hu et al., 2019). The Arabidopsis genome predicts five p300/CBP-type A HATs, one of which, PCAT2 (p300/CBP acetyltransferase-related protein 2), has HAT activity (Pandey et al., 2002). Specific HATs are responsible for the acetylation of different lysine residues in histones (Earley et al., 2007). H3K14 and H4K12 acetylation are catalysed by two GNAT class HATs, HAG1, and HAG2, respectively. HAM1 and HAM2 are MYST class HATs that redundantly acetylate H4K5. Multiple HATs are involved in the H3K9, H4K8 and H4K16 acetylations (Earley et al., 2007). On the other hand, the Type B HAT proteins are present in the cytoplasm. They catalyse acetylation of histone H4 at lysine 5 and 12 in the cytoplasm, before integration of histone into newly replicated chromatin (Parthun et al., 1996; Verreault et al., 1998). In maize, type B HAT functions as a heterodimeric complex and was detected in the cytoplasm as well as in the nucleus indicating its role in the nucleus also (Eberharter et al., 1996; Lusser et al., 1999). The number of HATs varies in different crops. For example, a tomato has 32 HATs while a litchi has only 6 (Aiese Cigliano et al., 2013; Peng et al., 2017).

Histone deacetylases have been categorised into three families based on sequence similarity and cofactor dependency: Reduced Potassium Dependency 3 (RDP3)/ Histone DeAcetylase 1 (HDA1), Silent Information Regulator 2 (SIR2) and the plant-specific Histone Deacetylase 2 (HD2) (Pandey et al., 2002). The catalytic domain of the SIR2 family (Sirtuins) requires NAD as a cofactor (Haigis and Guarente, 2006), whereas RPD3/HDA1 requires Zn^2+^ as a cofactor (Yang and Seto, 2007). Furthermore, HD2 isonly found in plants (Pandey et al., 2002). These have a histone deacetylase domain that is conserved and lacks a DNA binding domain (Kumar et al., 2021). Some of the HDACs have zinc finger motifs, which play a role in protein-protein interaction (Aquea et al., 2010; Peng et al., 2017; Kumar et al., 2018). HDACs also vary in different crops. In upland cotton, 30 HDACs were reported while in litchi there were only 11 (Peng et al., 2017; Kumar et al., 2018).

## Plant epibreeding

**FIGURE 2:**
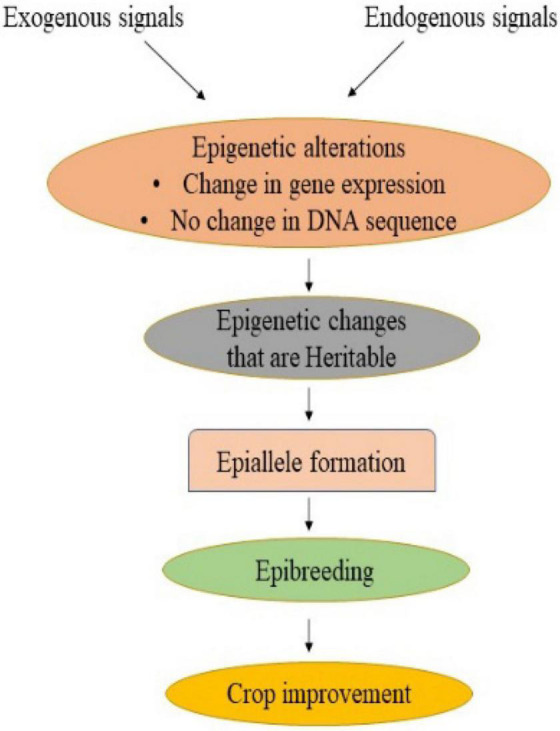
Epigenetic modifications can take place in response to external and internal cues, resulting in a change in gene expression without any change in DNA sequence. Stable and heritable epigenetic changes lead to the formation of epialleles which can be used in epibreeding programmes for crop improvement.

Traditional crop breeding involves crossing followed by the selection of genetic variants containing desirable traits (Palmgren et al., 2015). This resulted in a narrowing of the genetic base as well as a loss of genetic diversity, thereby hampering the crop improvement programme (Esquinas-Alcazar, 2005; Gallusci et al., 2017). Phenotypic traits, however, are influenced by both genetics and epigenetics. The exploitation of epigenetic variants or epigenome alteration could be a feasible breeding method for improving response to changing environmental conditions while ensuring agricultural yield and quality (Pecinka et al., 2010; Tirnaz and Batley, 2019). Many genes or QTLs governing traits of interest have been identified, but missing heritability remains a key barrier that affects phenotype. One of the key causes of missing heritability is epigenetic changes (Slatkin, 2009). The epialleles, also known as epigenetic alleles, are the loci having epigenetic modifications that may arise naturally or be induced artificially and are transferred stably to the next generation (Taudt et al., 2016). In addition to natural genetic variation, which contributes to phenotypic diversity, epialleles impart an additional source of heritable variation (Varotto et al., 2022; Figure 2). There are two ways in which epialleles could be formed: non-genetic (also known as epigenetic) and genetic (Taudt et al., 2016). Nongenetic epialleles originate from developmental factors or environmental signals that result in changes in chromatin state (Zheng et al., 2017). It also arises from spontaneous epimutation due to the inability to maintain an existing methylation state. The rate of spontaneous epimutations at cytosines in Arabidopsis has been observed to be 1,000 times higher as compared to the rate of genetic mutation (Becker et al., 2011). Besides, differentially methylated regions (DMRs), which span many cytosines, are substantially less common. It has been reported in soybean, maize, and tomato (Manning et al., 2006; Shen et al., 2018; Xu et al., 2019). Furthermore, genetic epialleles arise due to the insertion of a transposon in an intergenic region, leading to its inactivation primarily by methylation of DNA (Pecinka et al., 2013). The methylated DNA enhances methylation on all sides of the insertion site, leading to the formation of new epialleles (Varotto et al., 2022). It has been documented in Arabidopsis, rice and maize (Banks et al., 1988; Schmitz et al., 2013; Zhang et al., 2015).

Peloric mutants in toadflax (*Linaria vulgaris*) are a classical example of epialleles that are heritable. These have flowers with radial symmetry, whereas wild-type plants have bilateral floral symmetry. The epimutation is due to hypermethylation of the promoter region of the *Lcyc* gene, which is a CYCLOIDEA homologue of Antirrhinum and controls flower symmetry by silencing the gene (Cubas et al., 1999). An epiallele *clark-kent* (*clk*) was reported in Arabidopsis, which led to an increase in the number of stamens and carpels. This was due to hypermethylation of the cytosine base at the *SUPERMAN* locus, which was associated with flower development (Jacobsen and Meyerowitz, 1997). The switch from male to female flowers in melon was due to hypermethylation of the *CmWIP1* TF promoter region (Martin et al., 2009b). The epiallele *SP11*/*SCR* locus in Brassica was associated with self-incompatibility (Shiba et al., 2006). The epiallele of the *QSS* (QuaQuine Starch) gene in Arabidopsis arises due to methylation in the 5’ region and is responsible for starch metabolism (Silveira et al., 2013). It has been reported that 43 percent and 46 percent of epialleles are passed down in successive generations in two different lines of Arabidopsis (Hofmeister et al., 2017). Also, near-isogenic lines that separated from a common ancestor a century ago, displayed persistent methylome inheritance over generations despite variable environments (Hagmann et al., 2015). More than 99% of the methylome in maize and Arabidopsis is preserved between accessions (Li et al., 2015; Hofmeister et al., 2017). Thus, epialleles that are stably heritable across generations are of interest to breeders and can be utilised in crop improvement programmes. These can directly influence a plant’s phenotype. Epigenetic modifications can also be induced by chemical compounds. The methyltransferase inhibiting agents like 5-Azacytidine (5-AzaC), 5-Aza deoxycytidine, and Zebularine reduce methyl group transfer to cytosine and cause hypomethylation. Inhibitors of histone deacetylase include trichostatin-A (TSA), helminthosporium carbonum (HC) toxin, and nicotinamide, which enhance the acetylation of histones, thereby resulting in the activation of genes (Gahlaut et al., 2020). The transmission of induced epialleles has been reported in Arabidopsis in an epigenetic recombinant inbred line (epiRIL) population (Johannes et al., 2009; Reinders et al., 2009). EpiRILs are recombinant inbred lines derived from parents that have identical genetic makeup but they differ in DNA methylation (Johannes et al., 2009). These are produced by crossing mutants having hypomethylated DNA (*met1* and *ddm1*) with wild plants. The generated lines are homozygous for all traits except DNA methylation pattern, which affects traits like plant height, flowering time, biomass, yield and biotic as well as abiotic stress tolerance (Johannes et al., 2009; Reinders et al., 2009; Kooke et al., 2015; Zhang Y. Y. et al., 2018). It has been reported that the hypomethylated state was trans-generationally inherited in *A. thaliana*, even up to eight generations (Quadrana and Colot, 2016). Until now, two epi-RIL populations have been generated in Arabidopsis (Johannes et al., 2009; Reinders et al., 2009). The first one was produced by a cross of *met1* mutant (DNA methyltransferase defective) to its wild type (isogenic) (Kankel et al., 2003; Saze et al., 2003; Reinders et al., 2009). Another one was derived by a cross of a *ddm* mutant (*DDM* locus defective, which is responsible for maintaining cytosine methylation) to its wild type (isogenic) (Jeddeloh et al., 1999; Lippman et al., 2004; Johannes et al., 2009). A population with remarkably comparable genomes that differ only in methylation levels is produced by selecting progeny with the wild-type copy of a gene and then allowing them to self-pollinate for several generations. Some of these epiRILs can express information that is generally repressed by DNA methylation in natural variations due to the stripping of methylation at certain chromosomal locations (Kumar, 2019). The genes that are affected by DNA methylation can be mapped using epiRILs to determine their association with phenotypic traits (Cortijo et al., 2014; Kooke et al., 2015).

## Effect of epigenetics on agronomic traits in various crops

### Rice

Rice (*Oryza sativa* L.) is a key cereal crop that feeds more than half of the world’s population as a staple diet (Priya et al., 2019). The epiallele *epid1* codes for a GTP-binding protein, which causes stunted growth. The *d1* genes are silenced by DNA methylation together with histone acetylation, resulting in rice plant stature regulation (Miura et al., 2009). *OsSPL14* is an epiallele linked to WEALTHY FARMERS PANICLE (WFP). The epigenetic change enhanced *OsSPL14* expression, leading to an increase in panicle branching and grain yield (Miura et al., 2010). Flowering time (heading) is the most crucial stage for the production of grain in rice. Early flowering causes decrease in yield while delayed flowering results in a reduction in seed set. It is regulated by epigenetic modifications (He, 2009; Liu et al., 2010). Histone methyltransferase (HMTase) genes such as *SET DOMAIN GENE 724* (*SDG724*) belong to Class II in the SET domain family and promote flowering by methylating histone H3 lysine 36 (H3K36) (Ng et al., 2007; Sun et al., 2012). Under both long day (LD) and short day (SD), the *SDG724* loss-of-function mutant *lvp1* demonstrated delayed flowering, which was linked to decreased expression of *RICE FLOWERING LOCUS T 1* (*RFT1*) along with *Heading date 3a* (*Hd3a)*. Even though the two genes are near at a distance of 11.5 kb, only the chromosomal region of *RFT1* decreased the transcriptional activation through H3K36me2/3 modification (Sun et al., 2012). In another study, it was reported that the expression of *RFT1* can be enhanced in *Hd3a*-RNAi transgenic plants by acetylation of H3K9 near the start site of transcription (Komiya et al., 2008). *SDG725*, also belonging to SET domain family class II promotes flowering through H3K36me2/3 (Ng et al., 2007; Berr et al., 2009). OsTrx1, a member of Class III in the SET domain family, activates or conserves the active state of transcribed genes. It also prolongs the time of flowering in LD plants (Ng et al., 2007). *WOX11* (Wuschel-related homeobox gene) with the help of ADA2-GCN5 histone acetyltransferase regulates genes associated with crown root development (Zhou et al., 2017). A QTL *OsglHAT1* (a new-type GNAT-like protein) was reported to have inbuilt H4 histone acetyltransferase activity and was associated with grain weight. *PGL2* (*6-PHOSPHOGLUCONOLACTONASE 2*), which is critical for grain length, is positively regulated by *OsglHAT1*. Enhanced expression of *OsglHAT1* has a positive effect on agronomic traits like grain length, grain weight, yield, and total biomass (Song et al., 2015). In rice, OsSRT1 is a SIR2-type HDAC that suppresses carbon metabolic flux of the glycolysis pathway while enhancing the accumulation of starch in growing seeds (Huang et al., 2007; Zhang H. et al., 2016). Leaf angle is another agronomic feature that directly impacts the architecture of a plant and grain yield (Zhao et al., 2010; Jang et al., 2017). Plants having upright leaves catch more sunlight to carry out photosynthesis as well as have increased nitrogen storage for grain filling, thereby increasing yield and making them suitable for dense planting (Sakamoto et al., 2006). Leaf angle, grain size and yield potential are regulated by brassinosteroid (BR) phytohormones (Zhang et al., 2014). When the BR biosynthesis genes are overexpressed, the leaves become less upright and have a considerable leaf inclination whereas in BR-deficient or BR-insensitive mutants, the leaves are erect (Yamamuro et al., 2000; Hong et al., 2005; Wu et al., 2008; Li et al., 2009; Tong et al., 2012; Sun et al., 2015). Zhang et al. (2015) identified epiallele *Epi-rav6* being associated with a larger lamina inclination and smaller grain size. This is due to hypomethylation in the promoter region of RAV6. The *OsPCF7* gene which encodes transcription factors family viz TCP, plays a role in the architecture of the plant. Increased expression of *OsPCF7* in transgenic rice seedlings enhances the height of shoot, length of root, number of roots, tillers and heading, resulting in an increase in grain yield per plant (Li et al., 2020). Promoter DNA methylation is predicted to silence *OsFIE1* in vegetative tissues. Ectopic *OsFIE1* expression, caused by an epimutation with loss of promoter DNA methylation, results in dwarfism, floral abnormalities and changes in H3K27me3 levels in hundreds of genes (Zhang et al., 2012). The hypomethylation of the *ESP* gene is associated with the regulation of panicle architecture. The plants exhibited short and dense panicles (Luan et al., 2019). The deletion of SE1 or impairment in the function of components of the repressor complex hinders histone deacetylation and H3K27me3 methylation in the *Eui1* region, resulting in the switching of chromatin from a closed to an open state, thus increasing *Eui1* transcription, reducing gibberellic acid and causes dwarf phenotype (Xie et al., 2018). NGR5 promotes tillering in response to increased nitrogen supply by facilitating the recruitment of PRC2 (Polycomb repressive complex 2) to restrict the expression of shoot branching-inhibitory genes via H3K27me3 (histone H3 lysine 27 trimethylation) (Wu et al., 2020). Hypermethylation of the *OsAK1* gene at the promoter region is associated with photosynthetic capacity (Wei et al., 2017). The *OsSPL14* gene has been reported to be regulated by microRNA and affects the branching of panicles and increased yield (Miura et al., 2010). miR160 affects auxin signalling by down- regulating *OsARF18* expression, thereby affecting growth and development in rice (Huang et al., 2016). miR172 targets AP2-like TFs and affects the development of flowers, flowering time and branching of panicles (Zhu et al., 2009; Lee et al., 2014; Wang et al., 2015). Down-regulation of the *OsLAC* gene, which produces laccase-like protein by osa-miR397, controls grain size and yield (Zhang Y. C. et al., 2013).

### Maize

Maize (*Zea mays* L.) is an important staple crop cultivated worldwide to meet human needs (Xiao et al., 2017). Barbara McClintock discovered transposon silencing in maize (Ravindran, 2012), which is an epigenetic process. It reduces transposition and genomic disruption by transposable elements (TEs). Active TEs are frequently associated with hypomethylation, whereas silenced TEs show significant levels of DNA methylation (Chandler and Walbot, 1986; Okamoto and Hirochika, 2001). Many processes in plants, including development regulation (He et al., 2011; Grossniklaus and Paro, 2014), the response to external stimuli (Eichten and Springer, 2015) and adaptation, is influenced by epigenetic regulation via DNA/RNA methylation, posttranslational histone modifications and ncRNAs (Chinnusamy and Zhu, 2009). The methylation of the *ZmMRP4* gene produces a lpa1-241 phenotype with a high amount of inorganic phosphate in the seed (Pilu et al., 2009). In another study, methylation of the *p1* gene (which is Myb-homologous and regulates red pigment biosynthesis) is associated with a reduction in pigmentation (Cocciolone et al., 2001). *HDA108* is a histone deacetylase, that regulates plant height, leaf development and fertility by reducing H3K9 dimethylation and increasing H3 and H4 histone acetylation in nuclei (Forestan et al., 2018). Downregulation and overexpression of the *HDA101* histone deacetylase gene affected the number of acetylated histones, resulting in morphological and developmental defects (Rossi et al., 2007). Interaction of *HDA101* with corepressors such as NFC103/MSI1 and SNL1/SIN3-like proteins causes hypoacetylation of lysine 5 in histone H4 (H4K5ac) on targeted genes without affecting their transcript levels (Varotto et al., 2003). It influences the size of the kernel by regulating the transfer of cell-specific gene expression and its excision causes hyperacetylation of histones in the target region without affecting transcript levels. Some inactive genes are also targeted by *HDA101* which are associated with hyperacetylation and enhanced expression (Yang et al., 2016). ZmGCN5 advances the development of endosperm during seed maturation by interacting with the adaptor protein ZmADA2 and the bZIP transcriptional factor ZmO2 (Bhat et al., 2004). DNA methyltransferase genes (*dmt102* and *dmt103*) are silenced by DNA demethylation, resulting in apomixis-like phenotypes (Garcia-Aguilar et al., 2010). The *Arabidopsis FLOWERING LOCUS T* (*FT*) gene is an ortholog of the *ZEA CENTRORADIALIS8* (*ZmZCN8*) gene in maize, which codes for a phosphatidylethanolamine binding protein of the *ZmZCN* family (Danilevskaya et al., 2008). In transgenic plants, the expression of *ZmZCN8* can lead to early flowering, but suppressing *ZmZCN8* using artificial microRNA causes late flowering (Meng et al., 2011). The *INDETERMINATE1* (*ID1*) gene codes for a monocot-specific zinc finger transcriptional regulator that activates *ZmZCN8* expression in mature maize leaves (Lazakis et al., 2011). Through epigenetic control in the immature leaf, the ID1 protein controls the transcription of *ZmZCN8* and promotes nucleosome remodelling (Mascheretti et al., 2013). *ZmZCN7*, which is very similar to*ZmZCN8*, is engaged in remodelling regulation of chromatin indicating that it may code for a different maize florigen (Mascheretti et al., 2015). The intake of nutrients from soil like nitrogen (N) and phosphorus (P) is also regulated. Plants growing in less nitrogenous environments activate an adaption process that involves differentially produced miRNAs (Zhao et al., 2013). The miR169, miR399, miR408, and miR528 act in response to severe low nitrogen stress while miRC10 and miRC68 may respond to low soil nitrogen. In the case of a shortage of nitrate, miR528s, miR169s, miR166s and miR408/b cause developmental changes in roots (Xu et al., 2011; Trevisan et al., 2012; Zhao et al., 2012, 2013). miR169s, miR160f-5p, miR156s, and miR171s act by targeting NFY, ARF, SBP, and GRAS transcription factors, respectively, on exposure to cadmium stress (Wang et al., 2019). miR156 negatively regulates SBP-box transcription factor *tasselsheath4* (*tsh4*), allowing the formation of lateral meristems as well as inhibition of initiation of leaves and hence plays a vital role in establishing meristems and the boundary of leaves (Chuck et al., 2010). miR164 negatively regulates *NAC1* expression, which influences lateral root growth (Li et al., 2012). The expression of miRNAs can show significant differences during maize seedling development. The majority of miRNA target genes engaged in this mechanism are involved in transcriptional control. miR156, miR396, miR393, miR393 target *SPL*, *GRF*, *TIR1*, *LAC*, respectively, and may play a crucial role in the regulation of grain filling by governing growth and development (Jin et al., 2015). miR164 plays a regulatory role during seed development (Zheng et al., 2019). The development of the maize kernel is regulated by nine miRNAs belonging to the conserved zma-miR169 family (Xing et al., 2017).

### Tomato

Tomatoes (*Solanum lycopersicum* L.) are a globally important crop cultivated worldwide and are one of the most consumed vegetables (Kim et al., 2021). The silencing of the *MutS HOMOLOG1* (*MSH1*) gene by RNAi affects growth and development. These are due to DNA methylation as the observed phenotypes get reversed on treatment with 5-AzaC, which is a methylation inhibitor (Yang et al., 2015). The graft-mediated epigenetic changes are determined using the *MSH1* system in the rootstock. The *msh1* rootstock mutants have hampered siRNA formation. The progenies have enhanced growth vigor that is heritable over five generations and is RdDM-dependent (Kundariya et al., 2020). Hypermethylation of the promoter region of the colourless non-ripening (*cnr*) gene at CG and CHG regions silences the *cnr*, resulting in ripening defects (Manning et al., 2006). In wild fruits during ripening, a similar region is demethylated. Zhong et al. (2013) observed a loss of 5mC in the promoters of more than 200 ripening-related genes in a genome-wide investigation of DNA methylation in tomatoes. A decrease in DNA methylation is associated with the suppression of genes responsible for ripening, which also includes genes that participate in photosynthesis as well as the organisation of the cell wall (Lang et al., 2017). The DEMETER-like DNA demethylase (DML) *SlDML2* plays a critical role in controlling DNA methylation in ripening tomatoes. *SlDML2* inhibition by RNAi caused ripening defects, which were linked to increased methylation in the promoter region that supresses genes responsible for ripening and softening of fruit (Liu et al., 2015). Numerous differentially methylated genes in ethylene or carotenoid pathways encode transcription factors and important enzymes that may be targeted by differently produced non-coding RNAs. *ACO2* was targeted by MSTRG.59396.1 and miR396b, *CTR1* by MSTRG.43594.1 and miR171b, *ERF2* by MSTRG.183681.1, *ERF5* by miR9470-3p, *PSY1* by MSTRG.95226.7, *ZISO* by 12:66127788| 66128276, and *NCED* by MSTRG.181568.2 (Zuo et al., 2020). Fruit softening is influenced by histone post-translational changes and chromatin structural remodelling. To regulate tomato ripening, several genes producing histone deacetylases have been found, albeit genes from different subfamilies may play diverse roles. SlHDA3 and SlHDA1 from the RPD3/HDA1 subfamily, suppress genes involved in cell wall metabolism thereby acting as negative regulators of fruit softening. SlHDT3 of the HD2 subfamily, on the other hand, performed a beneficial function in fruit softening regulation by activating a similar set of genes that are regulated by SlHDA1/3 (Guo et al., 2017a,b, 2018). SlERF.F12 [belongs to the ERF.F subfamily consisting of Ethylene-responsive element-binding factor-associated Amphiphilic Repression (EAR) motifs] along with TOPLESS 2 (TPL2) and histone deacetylases HDA1/HDA3, reduces histone acetylation marks H3K9Ac and H3K27Ac at the promoter region of ripening related genes, resulting in transcription repression (Deng et al., 2022). The integration of a SINE retrotransposon in the promoter and hypermethylation of the inserted SINE has been linked to decreased Vitamin E (*VTE3*) gene expression (Quadrana et al., 2014). The upregulation of *VTE3* expression and an increase in fruit *VTE c*ontent is caused by spontaneous demethylation of the *VTE3* promoter (Quadrana et al., 2014). miRNA156 regulates plant height, leaf size and number as well as fruit size by targeting six SQUAMOSA PROMOTER BINDING PROTEIN (SBP)-box transcription factor genes (Zhang et al., 2011). miRNA156 also controls ovary and fruit development in tomatoes by targeting SPL transcription factors (Ferreira e Silva et al., 2014). miR157 modulates ripening in tomatoes by targeting the *LeSPL-CNR* gene, leading to mRNA degradation and translation repression. miR159 targets the *SGN-U567133* gene and plays a role in leaf and flower development. miR167 causes shorter petals, stamens and styles, as well as reduced leaf size and internode length by targeting ARF6 and ARF8 (Liu N. et al., 2014). miR4376 plays a role in the reproductive growth of tomatoes by targeting Ca^2+^-ATPase (Wang et al., 2011). miR168 causes phase transition, leaf epinasty and fruit development by targeting *AGO1* (Xian et al., 2014).

#### Soybean

Soybean (*Glycine max* (L.) Merr.) is an important seed crop. It supplies both protein and oil, mostly for feed and human consumption. The effect of epigenetics in domestication was investigated in 45 diverse accessions of soybean. Methylome analysis indicated that about 75% of DMRs were due to factors other than genetic variations (Shen et al., 2018). A breeding system for enhancing yield and stability in soybean was reported by Raju et al. (2018). By crossing wild-type and *MSH1*-acquired soybean memory lines, the *MSH1* system was exploited to create epi-lines with a wide variety for numerous yield-related parameters in both greenhouse and field trials. Additionally, obtained epitypes had little epitype–environment interaction, which indicated improved yield stability and less of an impact from the environment. The *MSH1* suppression-induced epigenetic variation can be passed down for at least three generations, and it can be bred for crop improvement through a few rounds of selection to improve and stabilise crop yield. DNA methylation is an evolutionarily conserved modification particularly in the symmetric CG context and causes the silencing of genes affecting leaf morphology, flowering time, floral organ identity, fertility and embryogenesis (Jacobsen et al., 2000; Miura et al., 2001; Choi et al., 2002; Xiao et al., 2006; Butenko and Ohad, 2011; Rea et al., 2012). Seed development is also affected by mutation in *met1* and *ddm1*, an ATP-dependent SWI2/SNF2 chromatin-remodeling factor (Lister et al., 2008; Lafos et al., 2011; Baubec et al., 2014; Hossain et al., 2017). Significant CG methylation in soybeans and DMRs occur between the methylomes of cotyledons, leaves, stems and roots (Song et al., 2013). An et al. (2017) reported role of DNA methylation during seed development. They observed methylation in CG (66%), CHG (45%), and CHH (9%) regions of cotyledons. CHH methylation levels increased dramatically from 6% in the initial stages to 11% in late stages. Also, transcribed genes were two times more differentially methylated than non-transcribed genes. In the CG, CHG and CHH contexts, 40, 66, and 2,136 genes were found with DMRs and a negative correlation between their expression as well as methylation was observed. Most of the *DMR* genes during seed maturation in the CHH context were transcriptionally down-regulated and were associated with DNA replication and cell division. In soybean seed coat, methylation in DNA was found to alter the transposition and splicing of a TE element from a MYB transcription factor that regulates anthocyanin synthase genes (Zabala and Vodkin, 2014).

### Rapeseed

Rapeseed (*Brassica napus* L.) is another principal oilseed crop and a major source of protein-rich livestock feed (Wang et al., 2020). Energy consumption efficiency is a distinctive characteristic of plant yield and vigour, and it has an epigenetic component that can be influenced by artificial selection. Populations with distinct physiological and agronomical traits were produced from an isogenic rapeseed population in which the individual plants and their self-fertilised progeny were repeatedly chosen for respiration intensity. Recurrent selection of isogenic lines of *B. napus* produces stable epigenotypes with better energy usage efficiency. Crossing these epigenetically different but genetically identical lines resulted in hybrids with a 5% yield enhancement (Hauben et al., 2009). Mutation in *BrSDG8* (which encodes a histone methyltransferase that affects H3K4 trimethylation in *FLOWERING LOCUS C* chromatin) is associated with early bolting that governs traits like leafy head formation and seed yield (Fu et al., 2020). In another study, insertion mutations in the *Bra032169* gene (encoding histone methyltransferase) are linked to early bolting (Huang et al., 2020). Recently, the role of Jumonji H3K27me3 demethylases (BraA.REF6 and BraA.ELF6) in controlling flower transition has been reported. *BraA.REF6* is more important than *BraA.ELF6* in regulating H3K27me3 levels. In *braA.ref6* mutants, the expression of *FLC* genes remains unchanged and there is a delay in flowering, while in *braA.elf6* mutants, H3K27me3 levels are high at *FLC*, leading to early flowering (Poza-Viejo et al., 2022). Through an epigenetic change of upstream *FLC* components, *BrcuHAC1* may influence the bolting and flowering time (Si et al., 2021). The demethylating drug 5-AzaC causes DNA hypomethylation in targeted regions, resulting in enhanced amount of seed protein and linoleic acid (Amoah et al., 2012). Solis et al. (2012) observed that decrease in methylation of DNA can initiate microspore embryogenesis. On the other hand, an increase in DNA methylation in a cell-type-specific manner is associated with pollen and embryo differentiation (Solis et al., 2012). 5-AzaC enhances microspore reprogramming, totipotency acquisition, and embryogenesis initiation and prevents embryo differentiation indicating role of methylation in the repression of microspore reprogramming and totipotency acquisition (Solis et al., 2015). A novel small molecule, BIX-01294, reduces histone H3K9 methylation thereby promoting microspore reprogramming and initiation of embryogenesis (Berenguer et al., 2017). During cell reprogramming and embryo development, H3K9me2 and HKMT are engaged in embryo cell differentiation and heterochromatinisation events, whereas H3Ac, H4Ac and HAT are involved in transcriptional activation, totipotency and proliferation events (Rodriguez-Sanz et al., 2014).

The improvement in agronomic traits through epigenetic modification in some other crops is given in Table 1.

**TABLE 1:** Epigenetic changes in crops associated with agronomic traits.

Crops	Trait/changes induced	Epigenetic modification	References
Cotton	• Methylation at H3K9me2 controls fibre differentiation by targeting synthesis of lipid and spatio-temporal modulation of reactive oxygen species	DNA methylation	Wang et al., 2016
	• Demethylation of DNA activates expression of COL2 gene which is responsible for photoperiodic flowering	DNA methylation	Hauben et al., 2009
	• Hypomethylation of DNA in non-embryonic calli stimulates plant regeneration	DNA methylation	Li J. et al., 2019
Poplar	• Demethylation of PtaDML10 (DEMETER-LIKE 10) gene causes breaking of buds and aids growth of shoot during chilling stress	DNA methylation	Conde et al., 2017
Apple	• DNA methylation at the MYB10 promoter regulates production of anthocyanin	DNA methylation	Telias et al., 2011
Sweet orange	• DNA hypermethylation of demethylase genes causes repression of genes involved in photosynthesis and cell wall organisation	DNA methylation	Huang et al., 2019
Sugarbeet	• Bolting tolerance due to methylation of DNA in genes associated with cold acclimation, hormonal pathway genes as well as flowering	DNA methylation	Hebrard et al., 2015; Trap-Gentil et al., 2011
Pineapple	• Demethylation of CpG islands in the promoter region of SERK1 is associated with enhanced somatic embryogenesis	DNA methylation	Luan et al., 2020
Pigeon pea	• Methylation in DNA is associated with heterosis	DNA methylation	Sinha et al., 2020
Wheat	• miR408 targets Timing of CAB Expression 1 TF and associated with heading time	mi RNA	Zhao et al., 2016
	miR159 targets MYB TF and is associated with development of anther	mi RNA	Wang et al., 2012
	cuticular wax biosynthesis by TaGCN5 and attenuation of a fungal pathogen	Histone modification	Kong et al. (2020)
Barley	miR172 targets APETALA2 (AP2)-like TF and is associated with grain density as well as cleistogamous flowering	mi RNA	Nair et al., 2010; Houston et al., 2013
	miR171 targets SCARECROW-like TF and is associated with phase transition as well as determination of floral meristem		Curaba et al., 2013
Potato	miR156 targets SQUAMOSA-promoter binding protein-like (SPL) TFs and is associated with plant architecture as well as tuber yield	mi RNA	Bhogale et al., 2014
	miR172 targets AP2-like TF and is associated with flowering time as well as tuberisation time	mi RNA	Martin et al., 2009a
Cotton	miR828 targets MYB TF and is associated with fibre development	mi RNA	Guan et al., 2014
	GhHDA5 is associated with fiber initiation	Histone modification	Kumar et al., 2018
Banana	Binding of MaHDA6 to the *MaERF11/15* promoters results in ripening	Histone modification	Fu et al., 2018
	MaHDA1 recruitment to target gene impeding ripening	Histone modification	Han et al., 2016

## Prospectives of crop epigenetics and epibreeding

Epigenetics contributes to phenotypic variation. Thus, understanding epigenetics and epigenomics can aid in elucidating the mechanisms through which environmental factors influence plant phenotypes (Agarwal et al., 2020). Breeders may be able to combat the ongoing issue of genetic erosion and uncover cryptic variation through the use of the existing epigenetic variability or epigenome modification. Epibreeding programmes could produce a wide range of phenotypic variability in just one generation and some of these alterations may be passed down from one generation to next (Dalakouras and Vlachostergios, 2021). It should eventually overcome all limitations and constraints that reduce the effectiveness of any crop breeding programme. Novel epialleles would be especially important in breeding populations with low genetic diversity (House and Lukens, 2019). As current breeding techniques mostly concentrate on genetics and disregard epigenetic factors, using epigenetic information at the epialleles level may provide new opportunities for crop development. However, further research on a wider variety of plant species is required to develop a more thorough knowledge of the processes that trigger and maintain epigenetic diversity in crops (Varotto et al., 2020).

## Conclusion

The development of high yielding crop varieties is the need of the hour so as to fulfil the demands of an ever-increasing population. Yield is a quantitative trait that is regulated by a number of loci as well as environment. Epigenetic mechanisms like DNA methylation, histone modification and chromatin remodelling have played a major role in controlling agronomic traits. These can increase the phenotypic variation that breeders can use to generate climate-resistant crops. There is also a need to capture and identify the epiallelic variations across the genome sequences of different crop species and even across species. The improvement in agronomic traits through epigenetics is found in many crops including rice, Arabidopsis, maize, soybean, rapeseed, etc. Existing breeding approaches generally focus on genetics and disregard epigenetic components, therefore using epigenetic information could bring new opportunities for crop development.

## Author contributions

Both authors contributed equally in writing and editing.
